# Dysfunction of apoptosis and autophagy correlates with local recurrence in esophageal squamous cell carcinoma after definitive chemoradiation

**DOI:** 10.1186/s12935-021-02171-9

**Published:** 2021-09-06

**Authors:** Hu Qiu, Haixia Song, Man Luo, Shaobo Ke, Wei Shi, Jiamei Chen, Wensi Zhao, Hesheng Luo, Yongshun Chen

**Affiliations:** 1 Department of Clinical Oncology, Renmin Hospital of Wuhan University, No.238 Jiefang Road, Wuhan, 430060 China; 2 Department of Radiation Oncology, Gansu Provincial Cancer Hospital, Lanzhou, China; 3 Department of Gastroenterology, Renmin Hospital of Wuhan University, No.238 Jiefang Road, Wuhan, 430060 China; 4 Department of Radiation Oncology, Zhengzhou University Affiliated Cancer Hospital, Zhengzhou, China

**Keywords:** Esophageal cancer, Chemoradiation, Recurrence, Apoptosis, Autophagy

## Abstract

**Objective:**

Definitive chemoradiotherapy (dCRT) is one of the standard treatments for esophageal squamous cell carcinoma (ESCC), but local recurrence is the main cause of treatment failure. The changes in apoptosis and autophagy in recurrent tumors of patients with ESCC following dCRT have been poorly estimated. Thus, this study aimed to investigate the expressions of key regulators of apoptosis and autophagy in matched paired samples of primary and recurrent ESCC.

**Methods:**

The medical records of patients with locally advanced ESCC who developed local recurrence after dCRT were reviewed, and the expression profiling of apoptosis-related genes, cell apoptosis, autophagy and autophagy-related proteins were detected in normal esophageal squamous epithelium and paired samples of primary and recurrent ESCC.

**Results:**

A total of 126 patients were enrolled, and 52.4% of them had stage III disease. The 1-, 3- and 5-year local recurrence-free survival (LRFS) rates were 54.8, 19.8 and 14.3%, respectively, with a median LRFS of 13.0 months. Patients with T2 tumor or stage II disease showed a significantly prolonged LRFS compared with that of patients with T3-4 tumor or stage III disease. The Apoptotic Machinery key genes expression profiling identified 5 upregulated and 7 downregulated apoptosis-related genes in recurrent tumors compared with their expression levels in the matched primary ESCC tumors. High expression of CD40, TRAF4 and BCL2A1, and low expression of CARD6 and TNFRSF21 were associated with increased risk of early local recurrence after dCRT. No differences in apoptotic index between primary and recurrent samples were detected. However, typical morphological features of autophagosomes and elevated LC3-II protein expression were detected in recurrent tumor samples, and positive LC3-II expression was correlated with increased risk of early local recurrence.

**Conclusion:**

Our findings indicated that apoptosis and autophagy dysfunction correlated with early local recurrence in patients with locally advanced ESCC receiving dCRT. Further studies are necessary to understand the biology of tumor recurrence in esophageal cancer.

## Introduction

Squamous cell carcinoma (SCC) and adenocarcinoma are the two predominant subtypes of esophageal cancer. Previous studies [1, 2] have investigated the effect of histological subtype on tumor molecular profile, and found that esophageal SCC (ESCC) is different from esophageal adenocarcinoma. Esophageal adenocarcinoma tumors are similar to gastric adenocarcinoma, but different from ESCC. ERBB2, VEGFA, GATA4 and GATA6 are the most commonly amplified genes in adenocarcinoma, whereas SCC has frequent genomic amplifications of CCND1, SOX2 and TP63. The CROSS trial [3], the SCOPE1 trial [4] and the study by Klevebro et al. [5] demonstrated that patients with ESCC could have more benefits from concurrent chemoradiation (CRT) compared with those with esophageal adenocarcinoma. However, local control after definitive CRT (dCRT) therapy for esophageal cancer remains a major problem, and locally recurrent lesions are mostly in the gross tumor volume, where the tumor burden is at largest [6]. Simultaneous integrated boost intensity modulated radiation therapy technique [7] and proton-beam therapy [8] could both effectively increase the radiation dose to the primary tumor. Targeting cell signaling pathways that increase the therapeutic ratio of radiation by sensitizing tumor tissues is another potential method of overcoming CRT resistance in esophageal cancer.

Apoptosis and autophagy are two important cellular processes associated closely with cell survival and cell death. A recent study revealed [9] that several apoptotic proteins modulate autophagy, and that autophagic proteins that control nucleation and elongation regulate intrinsic apoptosis. As a result, the interplay between apoptosis and autophagy serves an important role in cancer progression and treatment. Chemotherapy and radiotherapy should effectively induce cell death in cancer cells to overcome therapeutic resistance. However, esophageal cancer cells can use autophagy to cope with the stress caused by anticancer therapeutics through degradation and recycling of injured or aged proteins and organelles [10, 11]. To date, limited information has been revealed on the changes in cell apoptosis and autophagy in ESCC associated with local recurrence after sCRT. This study aimed to investigate the potential reasons by analyzing the expression of apoptosis and autophagy-regulatory proteins in paired primary and recurrent ESCC samples.

## Materials and methods

### Study population

The medical records of 1012 patients who were diagnosed with locally advanced ESCC and completed dCRT from March 2002 to September 2012 were reviewed. According to the National Comprehensive Cancer Network guideline, patients received radiotherapy via high-energy (≥ 6 MV) linear accelerators with three-dimensional conformal technique to plan the target volume. A total dose of ≥ 50.4 Gy was administered with standard fractionation (1.8 Gy/day for 5 days per week). For chemotherapy, patients received cisplatin (25 mg/m^2^ intravenous infusion) on days 1–3 and 5-FU (750 mg/m^2^ as a continuous intravenous infusion daily for 24 h) on days 1–4. The regimen was repeated every 4 weeks for 2–4 cycles.

A total of 126 patients who developed local recurrence, as confirmed by computed tomography, endoscopic ultrasound and biopsy, were enrolled in this study. The baseline characteristics of the patients are summarized in Table 1. After confirmation by pathologists, 126 pairs of primary and local recurrent ESCC samples (i.e., 252 ESCC samples of which 126 were from the primary tumor and 126 were from the paired recurrent tumor) were obtained in the form of paraffin-embedded.

**Table 1:** Baseline characteristics and analysis of prognostic factors for LRFS in 126 patients

Factor	Number (%)	Median (months)	1-year LRFS (%)	3-year LRFS (%)	HR	95% CI	P
Sex							
Male	90 (71.4)	13.7	58.9	20.0	1.23	0.83 to 1.83	0.296
Female	36 (28.6)	10.6	47.2	19.4			
Age at diagnosis (years)							
< 60	58 (46.0)	15.5	67.2	22.4	1.33	0.94 to 1.90	0.108
≥ 60	68 (54.0)	10.6	45.6	17.6			
Tumor location							
Upper thoracic	53 (42.1)	13.0	54.7	22.6	1.12	0.87 to 1.46	0.515
Middle thoracic	55 (43.6)	14.0	56.4	21.8			
Lower thoracic	18 (14.3)	9.0	50.0	16.7			
Differentiation							
Well	29 (23.0)	13.7	65.5	20.7	0.97	0.76 to 1.25	0.677
Moderate	68 (54.0)	12.5	51.7	27.6			
Poor	29 (23.0)	12.1	51.5	16.2			
T stage							
T2	58 (46.0)	15.5	67.2	29.3	1.45	1.14 to 1.85	0.007
T3	46 (36.5)	10.1	43.5	15.2			
T4	22 (17.5)	10.5	45.5	13.6			
Lymph nodes							
Negative	46 (36.5)	13.0	58.7	21.7	1.14	0.79 to 1.64	0.493
Positive	80 (63.5)	12.5	52.5	18.8			
7th AJCC stage							
II	60 (47.6)	19.0	63.3	31.1	1.85	1.27 to 2.69	0.001
III	66 (52.4)	10.6	47.0	10.6			

*LRFS* local recurrence-free survival, *HR* hazard ratio, *CI* confidence interval, *AJCC* American Joint Committee on Cancer

The institutional review board and ethics committee of Renmin Hospital of Wuhan University approved the present study, and informed patient consent was waived by the review board due to the retrospective nature of the study.

### Expression profiling of apoptosis-related genes

The commercially available Human Apoptosis RT2 Profiler™ PCR Array was used in this study. The array consisted of 96 primers for 84 protein-encoding Apoptotic Machinery (AM) genes and 5 housekeeping genes. Total RNA was extracted from tumor tissues using TRIzol reagent (Invitrogen; Thermo Fisher Scientific, Inc.) according to the manufacturer’s protocol. RNA samples were subjected to 1.2% agarose gel electrophoresis and purified with the StrataPrep Total RNA Microprep kit (Stratagene; Agilent Technologies GmbH). The SMART PCR cDNA Synthesis kit and Advantage 2 PCR kit (Thermo Fisher Scientific, Inc.) were used to verify the quality of the RNA samples, and cDNA synthesis was conducted with the High Capacity RNA-to-cDNA kit (Applied Biosystems; Thermo Fisher Scientific, Inc.). Reverse transcription-quantitative PCR (qPCR) expression profiling was applied to analyze 84 apoptosis-related genes in tissue samples from the primary and recurrent lesions in the same patient. Samples of normal esophageal squamous epithelium were manipulated similarly for negative control arm.

The data were analyzed using the 2^−ΔΔCq^ introduced by Livak and Schmittgen [12]. Standardization of the signal intensity between two arrays was based on the overall value of all genes on the arrays. The quantification cycle (Cq) for every gene in each sample was standardized to the Cq of the control genes according to the 2^−ΔΔCq^ method. To calculate the differential gene expression, a twofold threshold value was used for up- or downregulated genes having a fold change in expression levels ≥ 2.

### Detection of apoptotic cells

TUNEL staining was used to detect the apoptotic cells. Serial 4-μm-thick sections were deparaffinized in xylene and dehydrated in graded ethanol. The sections were incubated with 20 μg/ml proteinase K for 15 min and washed twice with 10 volumes of PBS for 10 min. The sections were incubated with H_2_O_2_ solution for 30 min to quench the endogenous peroxidase and rinsed with PBS. This procedure was performed according to the manufacturer’s protocol.

### Autophagosome visualization by transmission electron microscopy

Tumor cells were obtained by trypsinization in paraffin-embedded tissues, washed with PBS and fixed in ice-cold 3% glutaraldehyde buffered at pH 7.4 with 0.1 M cacodylate for 24 h. The cells were then fixed in 1.0% OsO_4_, dehydrated in a progressive ethanol and acetone solution, and embedded in Epon 812 in sequence. Sections (1-μm thick) were cut and stained with uranium tetraacetate followed by lead citrate trihydrate. Subsequently, the sections were observed with a H-600 IV transmission electron microscopy and photographed.

### Immunohistochemical staining for LC3-II

Serial 4-μm-thick sections cut from paraffin-embedded tissues were placed on silanized slides for immunostaining analysis. LC3-II protein expression was detected with a rabbit polyclonal antibody (1:800; Santa Cruz Biotechnology, Inc.). After deparaffinization, the tissue sections were dehydrated in graded ethanol, cleared in xylene and incubated with 3% hydrogen peroxide in methanol for 30 min to block endogenous peroxidase activity.

After overnight incubation with the primary antibody at 4 °C, the sections were washed twice in PBS and subsequently incubated with an anti-rabbit secondary antibody for 30 min. Diaminobenzidine and Mayer’s hematoxylin were used as substrate chromogen and counterstain, respectively. Normal esophageal epithelium was used as a negative control.

### Western blot analysis

As reported by Wolff et al. [13], 15 Sects. (4-μm thick) from the aforementioned formalin-fixed, paraffin-embedded blocks were used for protein extraction. Equal amounts of protein were boiled for 8 min in 1× loading buffer and subjected to western blot analysis using antibodies against LC3-II or actin according to the manufacturer’s protocols. An enhanced chemiluminescence reagent (Thermo Fisher Scientific, Inc.) was used to visualize the bands. The antibodies used were anti-LC3 (diluted at 1:500) and anti-β-actin, both from Santa Cruz Biotechnology, Inc.

### Statistical analysis

The data were presented as the mean ± standard error of the mean and analyzed by one-way ANOVA followed by post hoc Tukey’s test to determine the differences between groups. The recurrence interval was calculated from the end date of the dCRT to the date of first documented local recurrence. Survival probabilities were calculated by the Kaplan–Meier survival method. Differences in survival between groups were assessed using the log-rank test. The influence of each clinicopathological variable on survival was evaluated by multivariate Cox proportional-hazards regression model. The results of all models were reported as odds ratios with 95% confidence intervals (CIs).

All statistical computations were carried out using SPSS 22.0 software (IBM Corp.), and two-sided P < 0.05 was considered to indicate a statistically significant difference.

## Results

### Changes in the expression of genes related to apoptosis

**Fig. 1:**
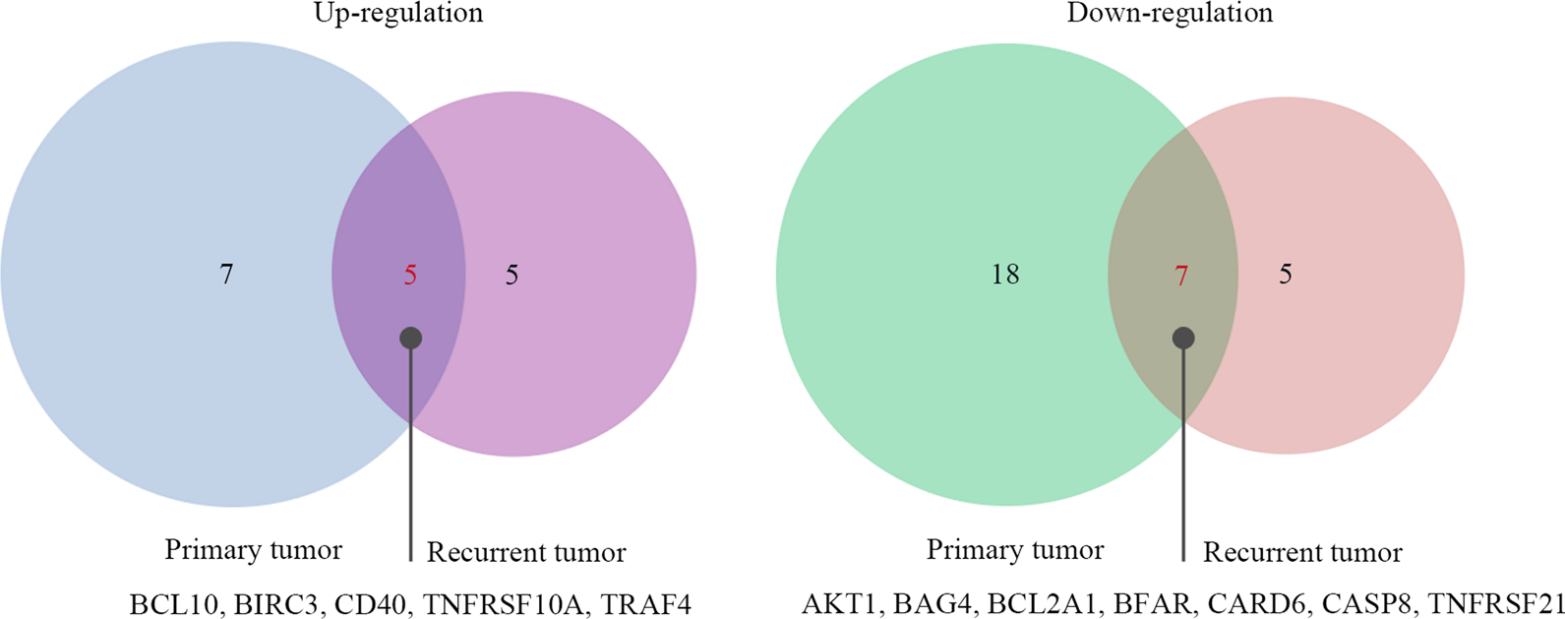
Venn diagram showing gene expression profile overlap between recurrent and paired primary tumor samples. Compared with the expression profiling of apoptosis-related genes of normal esophageal squamous epithelium in primary tumors and recurrent tumors tissue, the intersection analysis of up-regulated genes and down-regulated genes in primary tumors and recurrent tumors was conducted, and identical genes were found

Total RNA was extracted from the paired tumor samples and normal esophageal squamous epithelium. The total RNA concentration in 81 pairs of tumor samples varied from 381.5 to 762.6 ng/µl, with 260/280 ratios (optical density) of 1.91–2.03. The optical density of the remaining 45 pairs of samples was 1.51–2.01. Compared with that in normal esophageal squamous epithelium, 12 upregulated genes in primary tumor and 10 upregulated genes in recurrent tumor had an expression fold change > 2. Comparison between the pairs of primary and recurrent tumor samples revealed that 5 genes were concordantly upregulated with a > twofold difference in expression level, including BCL10, BIRC3, CD40, TNFRSF10A and TRAF4. Meanwhile, transcript quantification by the 2-ΔΔCq method demonstrated that 25 downregulated genes in primary tumor samples and 12 downregulated genes in recurrent tumor samples has a statistically significant difference in expression level (> -twofold) compared with that in normal esophageal squamous epithelium. The profiling data showed that 7 apoptosis-related genes were concordantly > twofold downregulated in recurrent tumor samples compared with the expression in paired primary tumor samples, including AKT1, BAG4, BCL2A1, BFAR, CARD6, CASP8 and TNFRSF21 (Fig. 1).

### Detection of apoptotic cells

**Fig. 2:**
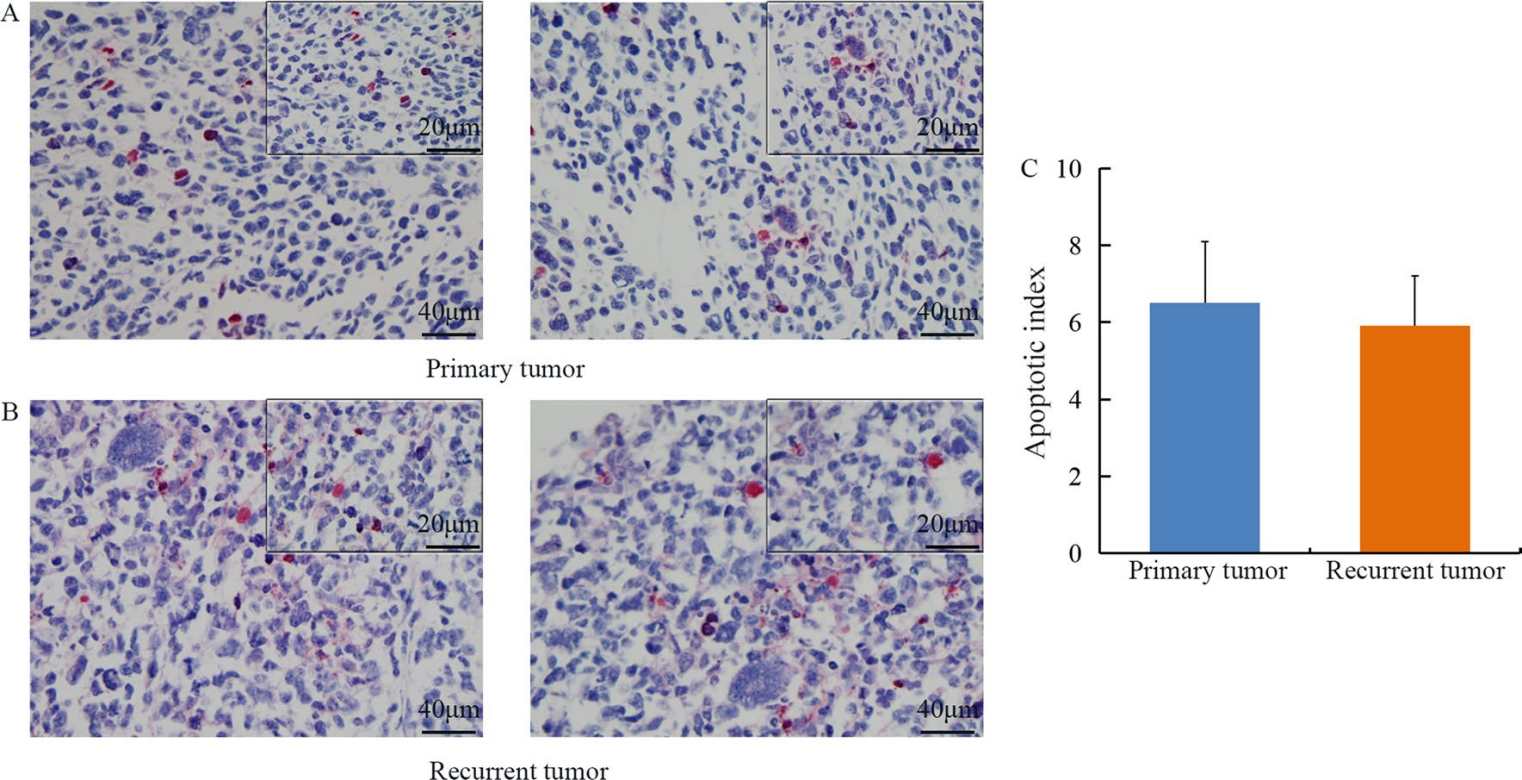
Apoptosis of tumor cells in primary and recurrent tumor samples. The apoptotic index is shown as the mean ± SD. Detection by TUNEL assay demonstrated a marked appearance of dark brown apoptotic cells in several primary (**A**) and recurrent (**B**) tumor samples. The apoptotic index was low in the two types of tumor samples, with no statistically significant difference between the two groups (P > 0.05) (**C**)

Detection by TUNEL assay demonstrated a marked appearance of dark brown apoptotic cells in several primary and recurrent tumor samples. However, the apoptotic index was low in the two types of tumor samples, specifically, 6.5 ± 2.7% in primary tumor samples and 5.9 ± 2.6% in recurrent tumor samples, with no statistically significant difference between the two groups (Fig. 2).

### Electron microscopy assay

**Fig. 3:**
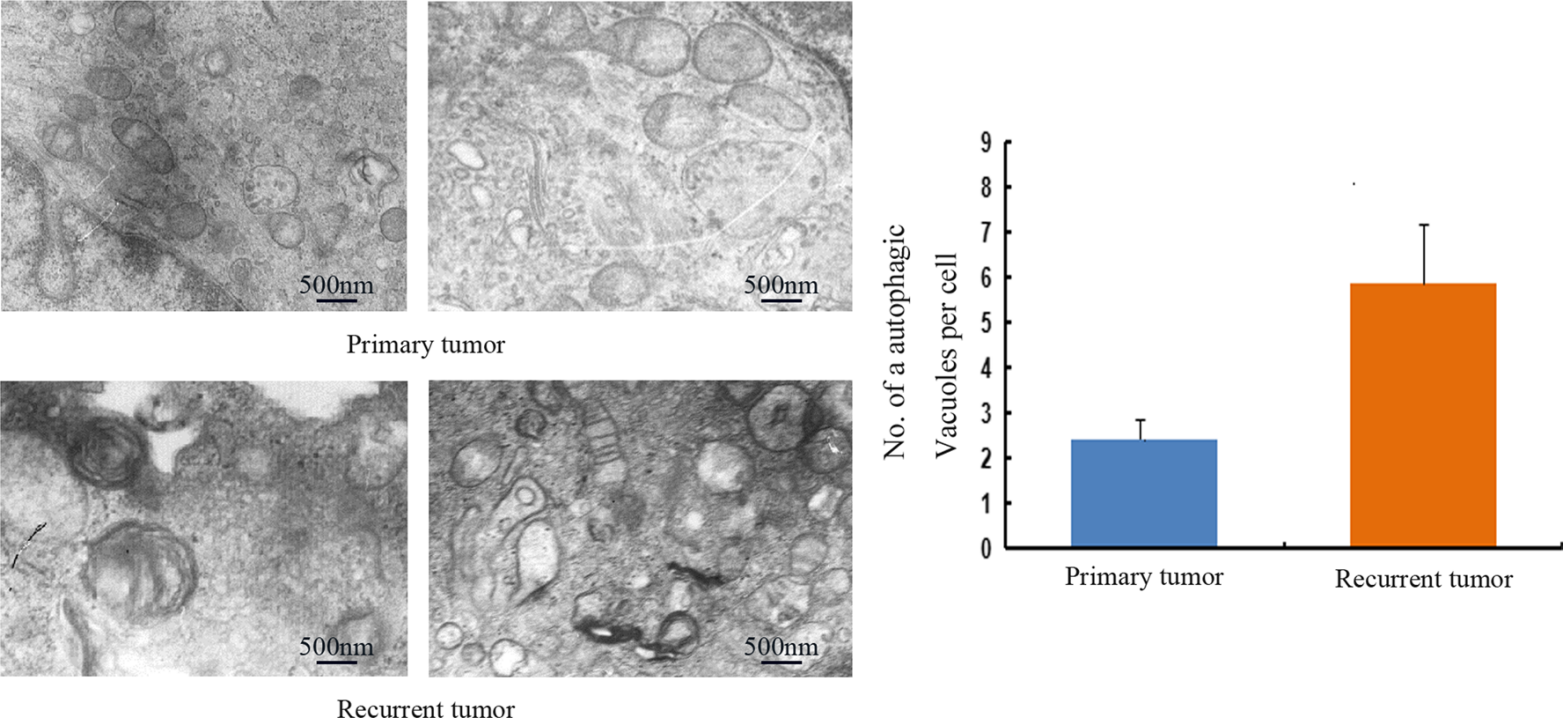
Autophagy in cells derived from primary and recurrent tumors. The numbers of autophagosomes per cell are shown. Data are presented as the mean ± SD. Subcellular structure analysis showed the typical morphological features of autophagy in cells from recurrent tumor samples, namely autophagic vacuoles containing cytoplasmic fragments or residual digested material. By contrast, only occasional autolysosomes were observed in cells from primary tumor samples (P < 0.05)

Transmission electron microscopy is the standard approach to reliably detect autophagy [14]. As shown in Fig. 3, subcellular structure analysis showed the typical morphological features of autophagy in cells from recurrent tumor samples, namely autophagic vacuoles containing cytoplasmic fragments or residual digested material. By contrast, only occasional autolysosomes were observed in cells from primary tumor samples. The numbers of autophagosomes per cell are shown in Fig. 3, 2.5 ± 0.8 in primary tumor samples and 5.8 ± 1.2 in recurrent tumor samples, with statistically significant difference between the two groups (P < 0.05).

### LC3-II protein expression analysis

**Fig. 4:**
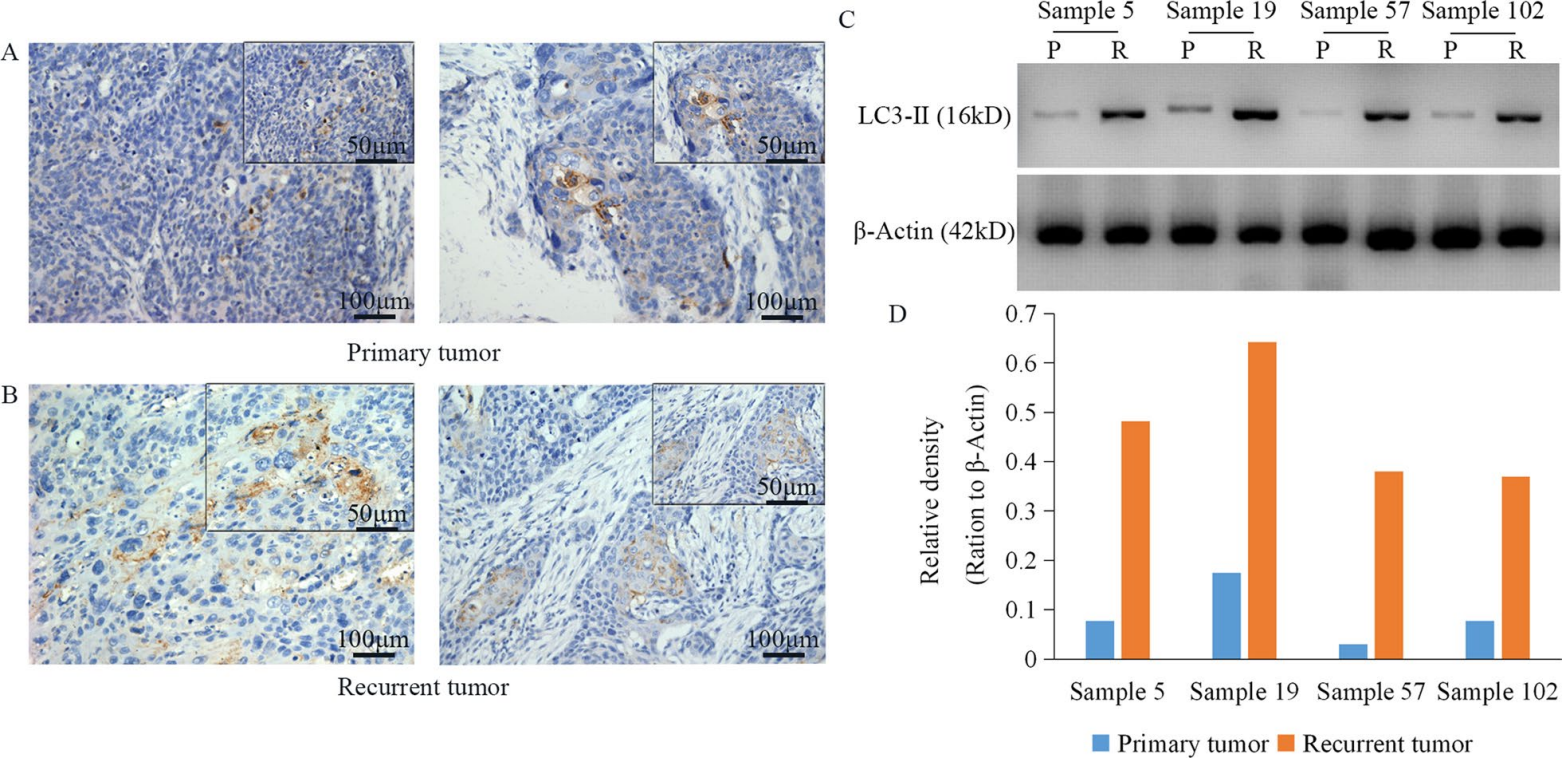
LC3-II protein expression in primary and paired recurrent tumor samples. Immunohistochemistry analysis of LC3-II level in primary (**A**) and paired recurrent (**B**) tumor. The numbers 5, 19, 57 and 102 refer to the sample numbers. Western blotting (**C**) analysis of LC3-II level in this paired tumor samples, integrated optical density quantification of LC3-II expression showed a statistically significant difference (**D**) (P < 0.05). P, primary tumor; R, recurrent tumor

The protein expression of LC3-II was evaluated by immunohistochemistry and western blotting. LC3-II staining was observed in the cytoplasm of esophageal cancer cells. Positive LC3-II-stained cells were noted with brown granules. LC3-II immunohistochemical staining was positive in 43.6% (55/126) of primary tumor samples and 54.8% (69/126) of recurrent tumor samples, with no statistically significant difference between the groups (P = 0.157; Fig. 4A). There was no discrepancy in LC3-II expression analysis between the two pathologists who evaluated the immunostaining. Protein bands immunopositive for LC3-II were clearly shown in each sample: Lanes 2, 4, 6 and 8 showed stronger bands for LC3-II in recurrent tumor samples, while primary tumor samples exhibited only a faint band for LC3-II (lanes 1, 3, 5 and 7). Integrated optical density quantification of LC3-II expression showed a statistically significant difference between the paired tumor samples (P = 0.002; Fig. 4B). There was 95.9% concordance rate in positive samples between the results of western blotting and those obtained by immunohistochemical staining.The concordance rate refers to that the positive samples of immunohistochemistry are basically consistent with western blot results, because some weak positive samples cannot be detected in immunohistochemistry, but can be detected in western bolt.

### Clinical and pathological factors predictive for local recurrence-free survival (LRFS)

**Fig. 5:**
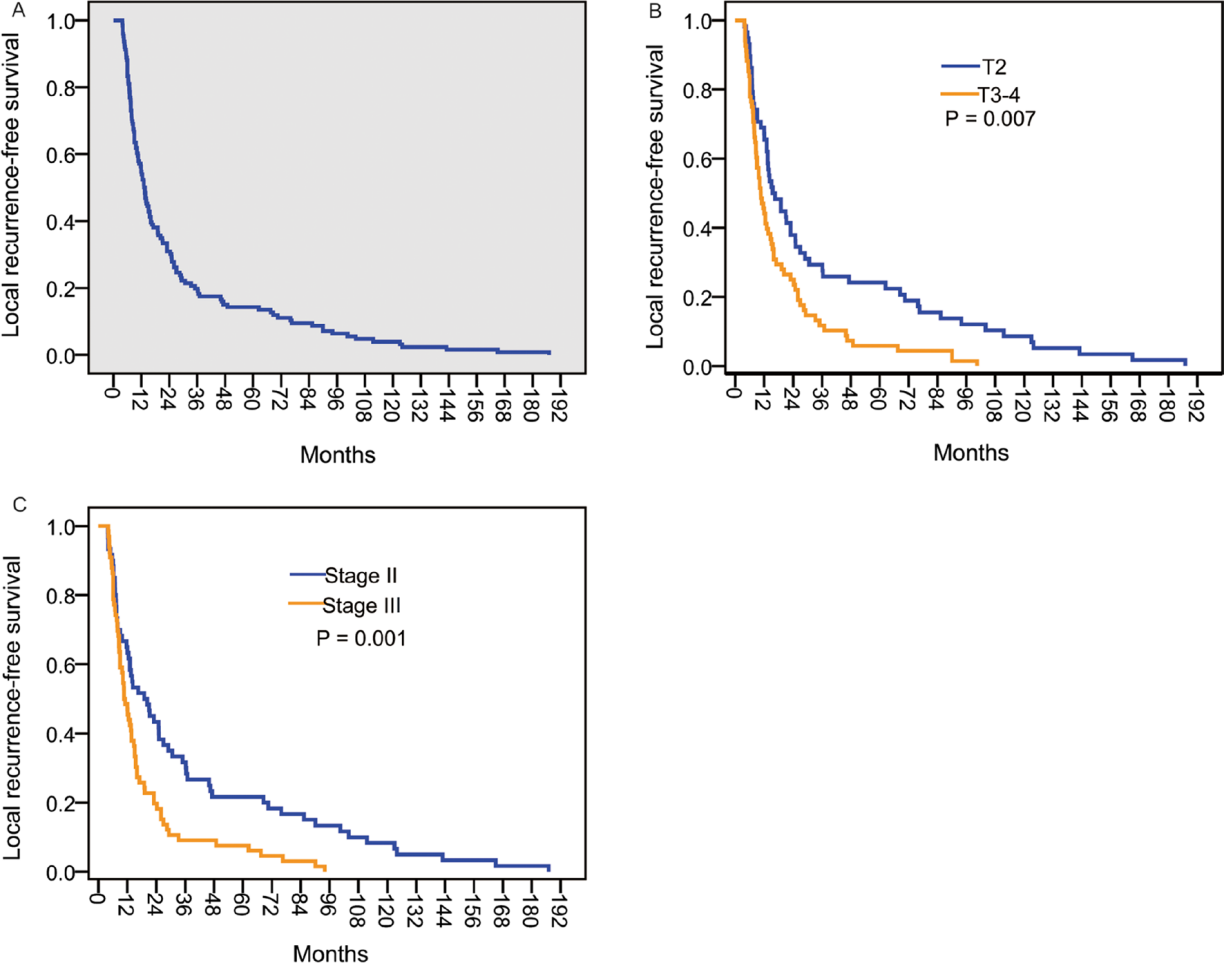
Kaplan–Meier Analysis of LRFS. **A** LRFS in the 126 patients enrolled in the present study. **B** Comparison of LRFS in patients with T2 vs. T3–4 stage. **C** Comparison of LRFS in patients with stage II vs. stage III disease. LRFS, local recurrence-free survival

The final date for follow-up was March 1, 2016. The median follow-up time was 56.5 months. A total of 15 patients were still alive during the final follow-up. For the total 126 patients, the 1-, 3- and 5-year LRFS rates were 54.8, 19.8 and 14.3, respectively, and the median LRFS was 13.0 months (95% CI, 10.9–15.1). The majority of patients (69.0%) developed local recurrence within 2 years after dCRT, while only 14.3% patients had local recurrence over 5 years after the initial treatment (Fig. 5A).

The risk factors of the patients and the tumor characteristics on LRFS were further investigated. As shown in Table 1, patients with clinical T2 stage showed significantly longer median LRFS than those with clinical T3-4 stage (15.5 vs. 10.1 months; P = 0.007; Fig. 5B). Patients with stage II disease had a median LRFS of 19.0 months, which was much longer than that of patients with stage III disease (i.e. 10.6 months; P = 0.001; Fig. 5C). Gender, age, tumor location, tumor differentiation and lymph node status had no significant correlation with LRFS.

### Correlation between expression level of apoptosis-related genes and LRFS

**Fig. 6:**
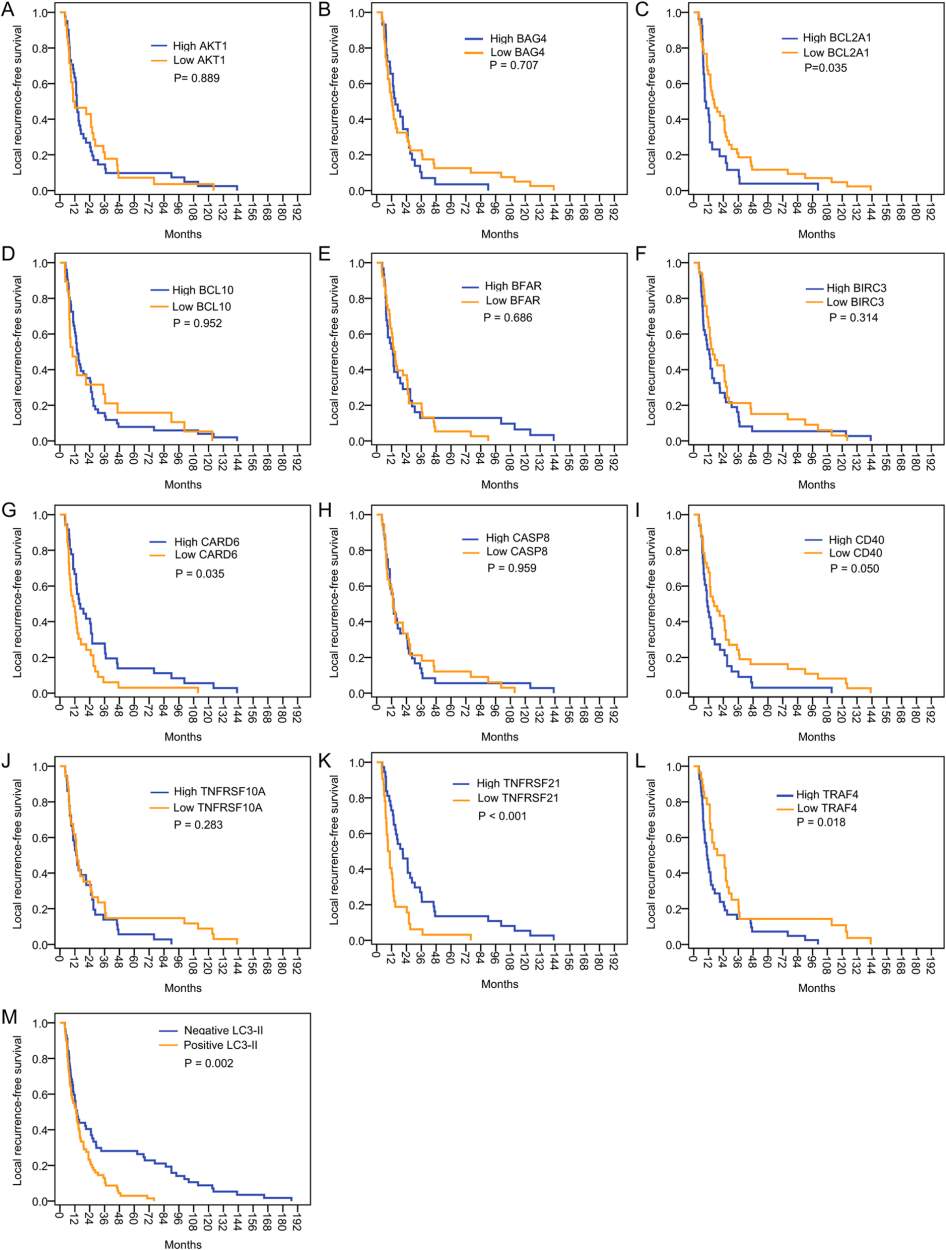
Kaplan–Meier Analysis of predictors of LRFS. **A** AKT1, **B** BAG4, **C** BCL2A1, **D** BCL10, **E** BFAR, **F** BIRC3, **G** CARD6, **H** CASP8, **I** CD40, **J** TNFRSF10A, **K** TNFRSF21, **L** TRAF4 and **M** LC3-II

The impact on LRFS of the 5 upregulated and 7 downregulated genes based on AM key genes expression profiling was investigated in 70 patients. According to the expression levels of the 12 apoptosis-related genes in the recurrent tumor samples, the patients were divided into two groups: High expression (≥ twofold upregulation or downregulation) and low expression (< twofold upregulation or downregulation). Kaplan–Meier plotter and the expression levels of the 12 genes are shown in Fig. 6A–L.

High expression levels of CD40, TRAF4 and BCL2A1 were associated with higher risk of early local recurrence after dCRT. Patients with ≥ twofold increase in CD40 expression had a shorter median LRFS than those with < twofold increase in CD40 expression (11.0 vs. 16.5 months; P = 0.050; Fig. 6I). Of the 42 patients with ≥ twofold increase in TRAF4 expression, 32 (76.2%) had local tumor recurrence within 24 months after dCRT. Out of 28 patients, 14 (50.0%) of which exhibited < twofold increase in TRAF4 developed local recurrence during the same period, and the median LRFS in the two groups was 10.6 and 19.0 months, respectively (P = 0.018; (Fig. 6L). Compared with patients that had a ≥ twofold increase in BCL2A1 expression, those with < twofold increase in BCL2A1 expression showed a significantly longer median LRFS, and the median LRFS in the two groups was 9.0 and 16.5 months, respectively (P = 0.035; Fig. 6C).

Oppositely, upregulation of CARD6 and TNFRSF21 in tumor samples was associated with lower risk of early local recurrence after dCRT. Patients with a ≥ twofold increase in CARD6 expression showed a significantly longer median LRFS compared with that of patients with a < twofold increase in CARD6 expression, and the median LRFS was 15.5 and 11.0 months, respectively (P = 0.038; Fig. 6G). Of the 37 patients with ≥ twofold increase in TNFRSF21 expression, 20 (54.1%) had local tumor recurrence, while 26 of 32 (81.3%) patients with < twofold increase in TNFRSF21 expression developed local recurrence within 24 months after dCRT, and the median LRFS in the two groups was 21.0 and 9.0 months, respectively (P = 0.001; Fig. 6K). The correlations between LRFS and the expression level of BCL10, BIRC3, TNFRSF10A, AKT1, BAG4, BFAR and CASP8 were not discovered in this study.

The correlation between the protein expression of the autophagy marker LC3-II and recurrence time was analyzed in patients with ESCC following dCRT. As shown in Fig. 6M, patients with negative LC3-II expression showed a median LRFS of 13.7 months, which was significantly longer than that of patients with positive LC3-II expression, with a median LRFS of 13.0 months (P = 0.002).

## Discussion

dCRT is considered as the standard of care in patients with esophageal cancer who are not surgical candidates. This therapeutic approach has a significant advantage of esophagus-preserving along with better quality of life. The CROSS [3] and SCOPE 1 trials [4] demonstrated that patients with ESCC could have more benefits from CRT compared with those with adenocarcinoma. However, patients with similar clinical and pathological features have different outcomes after receiving the same therapy, and while some are cured, while others experience local recurrence. Previous studies [6, 15] have investigated clinical and pathological factors that may be predictive of local recurrence in patients with esophageal cancer receiving dCRT, including tumor tissue, location, size and extent of the primary tumor. Those studies demonstrated lower rates of local recurrence in patients with SCC and with tumor located in the upper third of the esophagus. The local recurrence time interval was associated with T stage (T3/T4 vs. T1/T2; P = 0.002) and tumor size (> 8 cm; P = 0.009). In our study, T stage (T3-4 vs. T2; P = 0.007) and disease stage (stage III vs. stage II; P = 0.001) were correlated with early local recurrence in patients with ESCC after dCRT, while gender, age, tumor location, tumor differentiation and lymph node status were not.

Apoptosis and autophagy are correlated with oncogenesis, tumor growth and disease prognosis. It is necessary to investigate the expression of apoptosis and autophagy markers associated with different disease status. No previous study has been conducted to determine the prognostic values of certain apoptosis- and autophagy-associated factors for patients with ESCC receiving CRT. Human Apoptosis RT2 Profiler™ PCR Array was applied to analyze 84 AM key genes and 5 housekeeping genes in this study. The autophagy marker protein LC3-II was detected using immunohistochemistry and western blotting. Overall, 70 of 126 (55.6%) paired primary and recurrent tumor samples from paraffin-embedded tissues were screened, and 12 apoptosis-related genes with ≥ twofold difference in expression level were identified. The prognostic analysis showed that upregulation of CD40, TRAF4, BCL2A1 and LC3-II, and downregulation of CARD6 and TNFRSF21 were associated with higher risk of early local recurrence in patients with locally advanced ESCC who received dCRT.

The main goal of cancer treatment is to trigger tumor-selective cell death. Chemotherapeutic and radiotherapeutic agents should effectively induce cancer cell apoptosis to overcome resistance to antitumor therapy. Autophagy is a genetically programmed, evolutionarily conserved catabolic pathway. It has been regarded as a form of programmed cell death in some situation. Meanwhile, autophagy is a cytoprotective mechanism that assists cells to deal with a stressful metabolic environment and thereby promote cancer cell survival. O’Donovan et al. reported that esophageal cancer cells which were sensitive to the chemotherapeutic drugs 5-fuorouracil and cisplatin exhibited apoptosis, while chemoresistant cells exhibited autophagy. A significant increase in the expression levels of LC3-II was detected in chemoresistant esophageal cancer cells [10]. Autophagy was observed in esophageal cancer cells after exposure to ionizing radiation in our previous studies [11, 16]. Radiation therapy increased the protein expression level of Beclin-1 and the autophagy marker LC3-II in cell lines and xenografts, and the expression levels of the proliferative markers PCNA and Ki-67 were elevated. Our research group previously found that the level of LC3-II expression was correlated with the clinical stage, and the level of LC3-II expression in stage II patients was higher than that in stage III–IV patients [17]. However, high expression of LC3-II suggested a poor prognosis. In this study, the survival analysis showed that the patients with T2 and disease stage 2 had a better prognosis, so it may be that the patients with high expression of LC3-II in the early stage might have faster disease progression. Our study demonstrated that LC3-II immunohistochemical staining was positive in 54.8% of recurrent tumor samples, and patients with positive LC3-II had a shorter LRFS compared with that of patients with negative LC3-II expression. These indicated that esophageal cancer cells enhance their survival and recovery capabilities after chemotherapy and radiotherapy by induction of autophagy.

Another important determinant of resistance to anticancer treatment is dysregulation of different steps in the apoptotic pathways. The dysfunction may occur at the initiation and/or execution stages of apoptosis, which causes insufficient elimination of cancer cells and leads to resistance to treatment. The level of apoptosis index is related to the tumor progression, and the low level of apoptosis index in primary tumor samples was found in this study, which is the cause of local recurrence. At the same time, we also found that the apoptosis index of recurrent tumor samples was not significantly different from the primary tumor samples. Therefore, the key apoptosis-regulated genes in the recurrent tumor samples may be similar to those in the primary tumor. BIRC3 is a mediator of survival by allowing tumor cells to adapt to hypoxia. A previous study [18] reported that BIRC3 was a mediator of therapeutic resistance to standard chemotherapy and radiotherapy in glioblastoma. BAG-4 protein blocks signaling by preventing multimerization and subsequent activation of apoptosis, and loss of BAG-4 was observed to be associated with the development of resistance to cisplatin chemotherapy [19]. Cisplatin-based chemotherapy combined with radiation is the standard treatment for esophageal cancer. This study revealed that dysfunction of BIRC3 and GAG-4 might promote resistance to therapy in esophageal cancer cells.

AKT1, BCL2A1, BFAR, CARD6 and BCL10 are important apoptosis regulators. AKT1 inhibits apoptosis by phosphorylating and inactivating several targets, including caspase-9 [20]. CARD6 and BCL10 act as mediators for NF-κB, and they are closely associated with immune responses. Radiotherapy could alter the tumor microenvironment, mainly due to its effects on immune cells. The immune response elicited by radiotherapy might be as important as the radiation itself for tumor treatment. The correlations between the changes in the serum concentrations of IL-2 and IFN-γ during radiotherapy for esophageal cancer and treatment outcomes were investigated [21]. It was demonstrated that the serum concentrations of IL-2 and IFN-γ increased in patients who achieved complete or partial response, while they remained steady in those patients who had stable or progressive disease during radiotherapy. Thus, the intensity of the radiotherapy-elicited immune response was positively associated with local response to radiotherapy in esophageal cancer. Moreover, the dysfunction of apoptosis regulators might interfere with the immune response in patients with esophageal cancer receiving CRT.

Our study has certain limitations that should be acknowledged. Firstly, it was a retrospective study, and some data were not available in all patients, such as baseline albumin, weight loss during treatment, and changes in tumor markers, and leukocyte and lymphocyte counts. The missing information might influence the prognostic evaluation. Secondly, some patients enrolled in the study were treated over 10 years. It is well known that techniques for disease staging were markedly improved over these years. Besides, the DNA quality was insufficient for qPCR analysis in some long-term archived formalin-fixed and paraffin-embedded tissues. Third, the number of patients included in the study was small. Thus, selection bias could potentially affect the validity of the conclusion. Despite of these limitations, the results of our study are encouraging. Further investigation with a larger cohort size to explore additional apoptosis, as well as autophagy pathways and factors in tumor tissues is needed, which might provide further insight into the mechanisms of local recurrence in patients with ESCC after dCRT.
